# An integrated pore size distribution measurement method of small angle neutron scattering and mercury intrusion capillary pressure

**DOI:** 10.1038/s41598-021-97027-7

**Published:** 2021-08-31

**Authors:** Rui Shen, Xiaoyi Zhang, Yubin Ke, Wei Xiong, Hekun Guo, Guanghao Liu, Hongtao Zhou, Hang Yang

**Affiliations:** 1 Research Institute of Petroleum Exploration & Development, PetroChina Company Limited, Beijing, 100083 China; 2 China Spallation Neutron Source, Dongguan, 523803 China; 3 PetroChina Huabei Oilfield Company, Renqiu, 062550 China

**Keywords:** Characterization and analytical techniques, Crude oil

## Abstract

Small-angle neutron scattering and high-pressure mercury intrusion capillary pressure testing are integrated to analyze the pore size distribution of the broad sense shale oil reservoir samples of the Permian Lucaogou Formation in the Jimsar Sag, Junggar Basin, China. The results show that, compared with the measurement method integrating gas adsorption and mercury intrusion, combination of small-angle neutron scattering and mercury intrusion can more accurately characterize full-scale pore size distribution. The full-scale pore size distribution curve of the rock samples in the study area includes two types: the declining type and submicron pore-dominated type. The declining type is mainly found with silty mudstone and dolomitic mudstone, and most of its pores are smaller than 80 nm. Silt-fine sandstones and dolarenite are mostly of the submicron pores-dominated type, with most pores smaller than 500 nm. They also present large specific pore volumes and average pore diameters of macropores and are the favorable lithogenous facies for development of high-quality reservoirs.

## Introduction

Conventional oil and gas resources have been highly explored and exploited over the past years, and the remaining resources are declining. Unconventional oil and gas resources, represented by tight oil and shale oil, emerge rapidly and have become an important relay area for China's petroleum industry to increase reserves and stimulate production^1–3^. The nano scale ranges from near-atomic scale (nano) to near-optical scale (micron). In recent years, non-optical probing methods such as the diffraction method and electron microscopy have gradually replaced the optical microscopy and serve as the main tool for investigating nano-scale structures, which successfully raise the measurement precision of pore sizes from microns to nanometers^4^.

At present, the commonly used pore size distribution testing techniques include the fluid intrusion approach, e.g. the high-pressure mercury intrusion capillary pressure method (MICP), the gas adsorption approach (low-temperature N_2_ adsorption and the CO_2_ adsorption)^5–12^, and the non-intrusive approach, e.g. the small-angle neutron scattering (SANS)^13–17^.

Scholars in both China and other countries have extensively applied the gas adsorption method to investigate the properties of both the adsorbent and adsorbate, fractal dimensions, and pore types^18–23^, and this method is also combined with the fluid intrusion method to carry out systematic research on pores with diameters above 0.35 nm^24^. N_2_ adsorption and CO_2_ adsorption can only use powder samples for experiments, so it is necessary to fully consider the impact of sample particle sizes and water content. If the particle size is too large, the measurement of pores will be incomplete, while part of closed pores will turn into open pores in the case of excessively small particle sizes^25^. In the full-scale pore size characterization, the experimental temperature varies with different methods. The experimental temperatures of N_2_ adsorption, CO_2_ adsorption and MICP methods are respectively -196.15ºC, 0ºC, and the room temperature. Moreover, the MICP method is also able to provide not only pore diameter measurements but also information of pore-throat distribution. In terms of measurement merging, only measured data of parallel samples (thus not exactly the same sample) can be used. Therefore, the treatment of the data overlap remain considerably controversy.

SANS can analyze samples non-destructively at different temperatures, and simultaneously measure connected pores and closed pores in the sample, with high accuracy^26^. Using the SANS technique, Yang Rui et al. used the Porod invariant method and the PDSP model to calculate the pore size distribution, specific surface area and porosity of nanopores, and compared them with the results of the MICP method^27^. Subsequently, by studying the Longmaxi shale samples, it was found that the pore volume distribution presents a power-law pattern; the measured pore size distribution is consistent with the result of the N_2_ adsorption method in the same size range; the specific surface area and porosity of the PDSP method increase with growth of total organic carbon (TOC)^28^. Zhang Yuxiang et al. analyzed the Bakken shale by integrating SANS and MICP, which reflected the difference between the results of connected pores and total porosity, and with the help of the FE-SEM technology, identified the high contribution of organic pores to the total porosity^29^. Jitendra Bahadur et al. studied the microstructure of New Albany shale samples with different thermal maturities, and extracted information about the size range and number density of micropores from the relative fluorescence scattering intensity observed in the range of a large scattering vector Q. Moreover, they compared the results of the model-independent Porod invariant method with the calculation results of the PDSP model^30^. Sang Guijie et al. used SANS, N_2_ adsorption and MICP to study the nanopore structure characteristics of one clay and four shale samples. It was shown that the measured pore volume distributions are consistent in the range from one nm to several hundred nanometers, which demonstrates that micropores and mesopores have major contributions to the total pore volume. Furthermore, this research pointed out that the water absorption capacity of the sample is positively correlated with the total porosity, with the help of the dynamic water steam adsorption technique^31^.

The pore size span of shale oil reservoirs is very large, from less than one nanometer to sub-millimeters, and thus there is no test methods that are able to solely obtain the full-scale pore size and distribution characteristics of shale. Accurately capturing the characteristics of full-scale pore size distribution of shale is the basis of the micro-scale reservoir evaluation and have important guiding significance for studying the regularity of shale oil and gas occurrence. At present, the main testing methods for studying the pore size distribution of shale samples include CO_2_ adsorption, low-temperature N_2_ adsorption, SANS, and high-pressure MICP. The CO_2_ adsorption measures the pore size range below 2 nm, and the low-temperature N_2_ adsorption measures pores of 2–200 nm. SANS probes pores no more than 100 nm, and the high-pressure MICP (with test pressure up to 413 MPa) measure pores no smaller than 3.2 nm. CO_2_ adsorption, low-temperature N_2_ adsorption, and high-pressure MICP are commonly used to obtain the full-scale pore size of shale. However, this approach cannot measure the same sample because CO_2_ adsorption and low-temperature N_2_ adsorption require powder samples of 30–100 mesh, while high-pressure MICP can only accept cylinder or blocky samples, and the samples after MICP tests cannot be used for other experiments. Hence one shale sample has to be divided into two parts for different tests, and otherwise parallel samples will be used for measurement.

This paper proposes a SANS-MICP integrated measurement method for full-scale pore size distribution. Samples of the broad sense shale oil reservoir of the Permian Lucaogou Formation, the Jimsar Sag, Xinjiang, were used to carry out the quantitative analysis of pore size distribution characteristics, in an attempt to provide a novel idea and method for studying the pore size distribution of such broad-sense shale oil reservoirs.

## Materials and methodology

### Experimental samples

**Figure 1:**
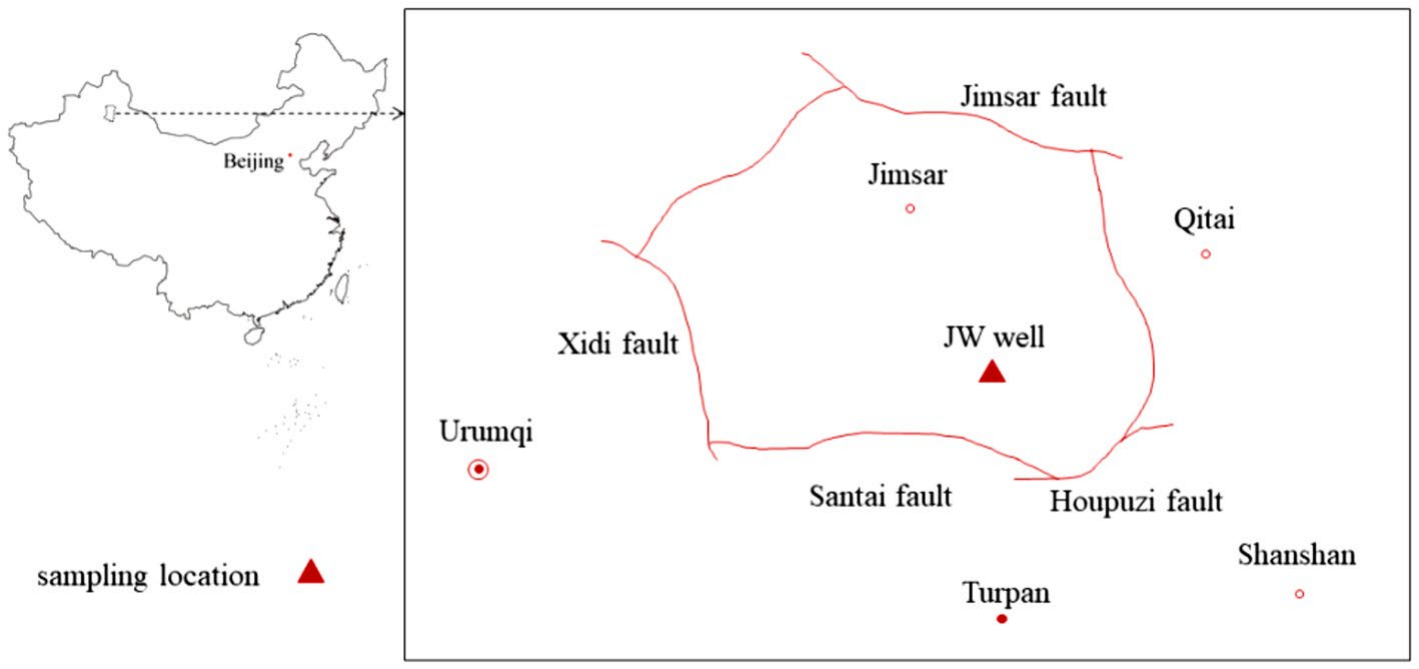
Geological map showing the sample location.

The core samples used in the experiment were collected from the Permian Lucaogou Formation broad sense shale oil reservoir in Jimsar, Xinjiang Uygur Autonomous region, China. The reservoir is located in the Jimsar Sag in the eastern Junggar Basin, 150 km away from Urumqi, as shown in Fig. 1.

**Figure 2:**
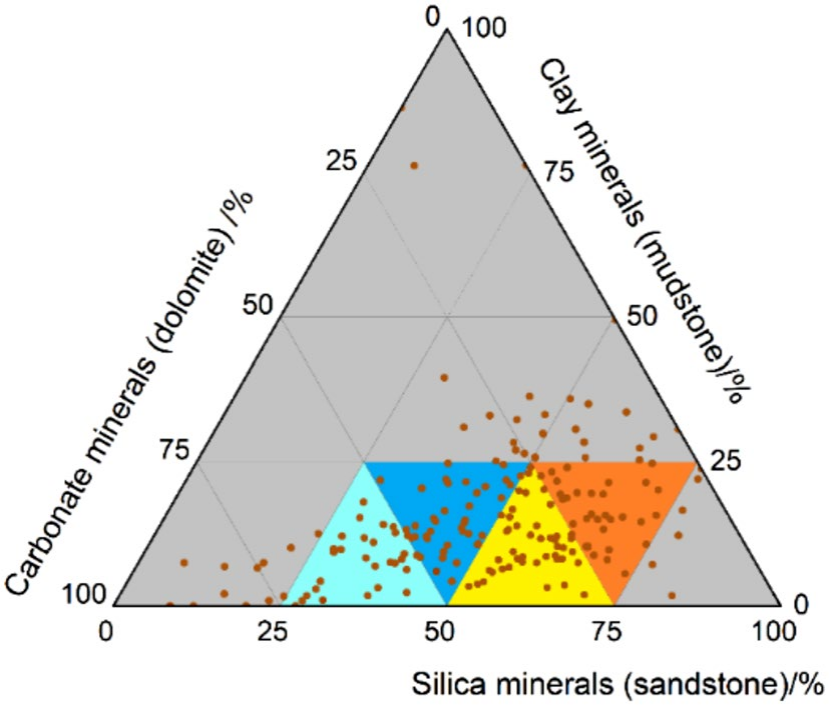
Ternary diagram of sandstone, mudstone and carbonate.

The geological structure, a monoclinal high in the east and low in the west, is stable. The main part of the formation has a dip angle of 3°–5°, and faults are not developed. According to physical properties and oil-bearing properties, the Lucaogou Formation vertically has two oil layer-concentrated sections, defined as “the upper and lower sweet-spot bodies”, within which there are multiple thin oil layers, with the single-layer average thickness of 0.32 m. TOC content of the sweet-spot reservoirs is generally greater than 1%, and the reservoir is alternatively interbedded by the source rock, which is considered favorable association. The upper sweet spot is the most important set of reservoirs in the Lucaogou Formation in the Jimsar Sag. The lithology is mainly dolarenite, feldspar lithic silt-fine sandstone, dolomitic mudstone, and silty mudstone, among which dolomitic mudstone is the main source rock. According to the results from X-ray diffraction (XRD) bulk rock analysis of more than 170 core samples, it is concluded that a diverse variety of minerals are present in the sweet-spot interval in the Lucaogou Formation, the Jimsar Sag, with the majorities being silt-fine grained sandstone, mudstone and carbonate rock. Silt-fine grained sandstone includes dolomitic silt-fine grained sandstone and feldspathic silt-fine grained sandstone, and carbonate rock is mainly dolarenite, as demonstrated in Fig. 2. Layers of silt-fine grained sandstone, shale, and dolarenite are all found with thickness at centimeters and demonstrable rhythms. These rocks experience heterogeneous mineral alterations such as silicification, dolomitization and albitization during diagenesis, which leads to complicated mineral composition, rapid vertical lithologic variation, and consequently limited thickness.

The core sample used in this experiment has average permeability of 0.0213 mD, average porosity of 9.96%, and an average density of 2.348 g/cm^3^. The basic physical properties of each sample are shown in Table 1. The S-Series samples are located in the orange area in Fig. 2, and the M-series samples are located in the light blue area in Fig. 2.

**Table 1:** Basic physical properties of core samples.

No.	Permeability (mD)	Porosity (%)	Density (g/cm^3^)
S6857	0.1046	11.42	2.377
S6819	0.0196	14.13	2.242
S6820	0.0420	15.36	2.205
M6859	0.0014	9.32	2.346
M6858	0.0006	5.98	2.418
M6822	0.0016	8.60	2.379
M6856	0.0001	10.37	2.339
M6860	0.0004	4.51	2.478
Average	0.0213	9.96	2.348

### Experimental methodology

For the SANS and high-pressure MICP tests, one core sample was divided into two parts. The SANS test obtains the specific pore volume distribution of pores with diameters ≤ 100 nm, while the MICP test obtains that of pores with diameters ≥ 3.4 nm. The MICP test, affected by the low mercury intrusion saturation when dealing with nano-scale pores, cannot cover all nano pores, and moreover high pressure may lead to occurring of secondary pores, which thus lead to inaccurate measurements. Therefore, in this research SANS was used to test the specific pore volume distribution of pores ≤ 100 nm, and MICP was used to measure the specific pore volume distribution of pores > 100 nm. Then the results of the two tests were merged to produce the specific pore volume distribution over the full pore size range. This procedure is a quantitative investigation method of full-scale pore-size distribution for shale reservoir core samples.

### Principles of SANS

Sans experiment was carried out in China spallation neutron source (CSNS) by small angle neutron scattering spectrometer. The wavelength of the incident neutron is 1–10 Å, and the distance between the sample and the detector is 4 m. The test time of each sample is about 120 min and the size of samples measured by SANS is 10 mm × 10 mm × 1 mm. Neutron scattering based on diffraction has been widely applied to material science. Neutrons interact with each other via short-range nuclear reactions and have very strong penetrating capability. Meanwhile they will not heat and damage the sample. Hence it is able to elaborately investigate the volume structure of the sample. The wavelength of neutrons and the wavelength of atoms are comparable in magnitude and spacing, and the energy of neutrons is equivalent to the energy of normal modes in the material (for example, phonons, diffusion modes), so they can be used for research on the dynamics of solid and liquid materials. Since the interactions of neutrons with hydrogen and deuterium are very different, deuterium labeling is a classic method in neutron scattering, which is mainly practiced by using deuterated molecules in a non-deuterated environment. The sub-coherent scattering lengths of hydrogen and deuterium are highly different, which helps to enhance the contrast of specific structural features.

**Figure 3:**
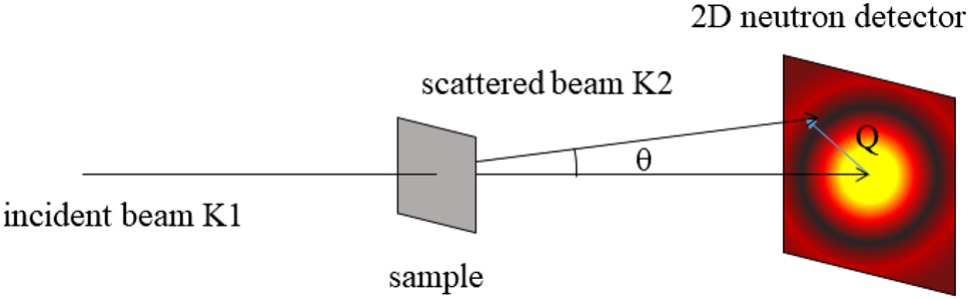
Schematic diagram of the experimental principle of SANS.

SANS is essentially caused by the variation of the scattering length density (SLD). SANS analyzes the micro structural of samples by measuring the intensity of neutron scattering occurring at small angles (scattering angles 2*θ* ≤ 5°) after long-wavelength neutrons penetrating the sample. In Fig. 3, K1 is the incoming beam and K2 is the scattered beam. Small-angle scattering specifically refers to scattering with a small *Q* value, and moreover the diffraction phenomenon must meet the Bragg condition, when a neutron beam passes through a crystalline material:

1 $$n\lambda = 2d\sin \left( \theta \right)$$

where, *d* is the distance between the lattice planes in meter; *θ* is the angle between the incident light source and the scattered light source in degree. When *n* = 1, the scattering vector formula *Q* = 2π/*d* applicable to crystalline materials can be obtained. For disordered porous shale with an average radius of *R*, *R* = 2.5/*Q*, which means that *Q* is dependent on the pore size of the sample. This is the theoretical basis for measuring the pore size distribution of rock samples via SANS.

The original SANS data was reduced to 1D absolute scale via IGOR Pro software^32^ (https://www.wavemetrics.com/order/order_igordownloads.htm). The data were analyzed using PRINSAS software^33^ (http://smallangle.org/content/software).

### Principles of high pressure MICP

**Figure 4:**
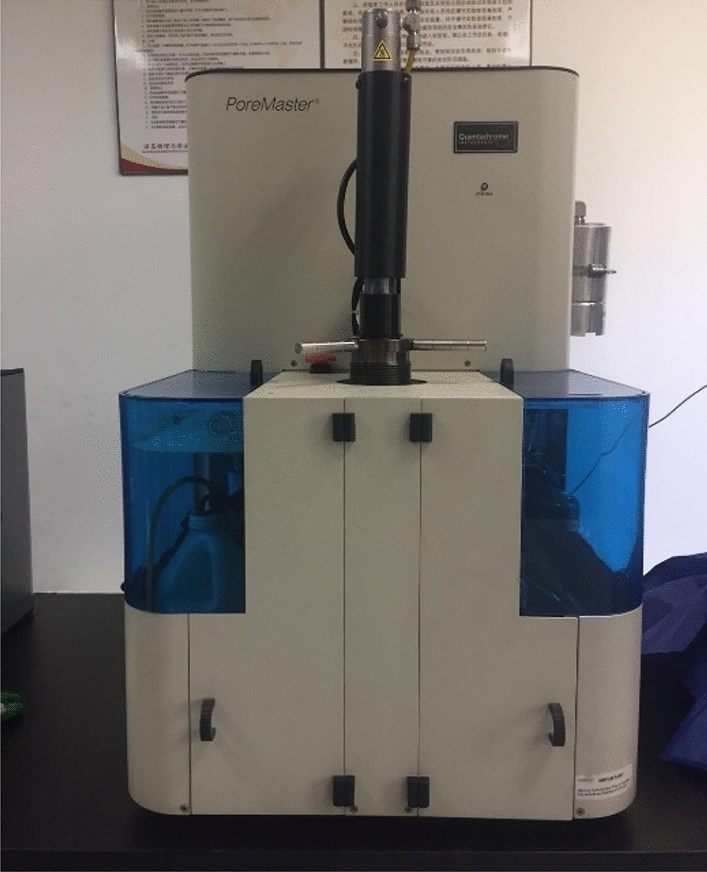
Poremaster high pressure mercury porosimeter.

MICP relies on the externally applied pressure to overcome the surface tension and inject mercury into pores so as to determine the pore size distribution. Increasing the applied pressure allows mercury to enter smaller pores, and thus more mercury injected into pores. Mercury is a non-wetting phase for general solids. To inject mercury into pores, an external pressure must be applied. The greater the external pressure is, the smaller the radius is, for pores that mercury can enter. By measuring the amount of mercury entering pores under different external pressures, the pore volume of the corresponding pore size can be known. The high pressure mercury intrusion experiment was completed by poremaster mercury injection instrument produced by Quantachrome instrument company (Fig. 4). The maximum applied pressure of the high-pressure MICP in this study is 413 MPa, and the measurable pore diameter ranges from 3.2 to 950,000 nm. The samples measured by SANS and MCIP are all from the same one sample, The sample measured by MCIP was made into a plunger with a diameter of 25 mm and a length of 15 mm.

The Washburn equation is used to calculate the pore size distribution of the shale pores ≥ 3.2 nm. The Washburn equation is shown below:

2 $$\Delta P = {\raise0.7ex\hbox{${ - 2\gamma \cos \theta }$} \!\mathord{\left/ {\vphantom {{ - 2\gamma \cos \theta } R}}\right.\kern-\nulldelimiterspace} \!\lower0.7ex\hbox{$R$}}$$

where ∆*P* is the pressure applied on the liquid surface, Pa; γ is the surface tension of the liquid, Pa; *θ* is the contact angle of the liquid, °; *R* is the pore radius, m. The surface tension of mercury equals to 0.48 N/m, and the contact angle between mercury and various substances is between 135° and 150°. Usually the average value of 140° is used for calculation, so the above formula can be simplified as:

3 $$\Delta P = {\raise0.7ex\hbox{${0.736}$} \!\mathord{\left/ {\vphantom {{0.736} R}}\right.\kern-\nulldelimiterspace} \!\lower0.7ex\hbox{$R$}}$$

Generally, the size of sans test sample is 10 mm × 10 mm × 0.5 mm to ensure neutron penetration and avoid multiple scattering. Therefore, in this research SANS was used to test the specific pore volume distribution of pores ≤ 100 nm。 In the future, the extension of Q-range can be completed by ultra small angle neutron scattering (USANS), which has reached a larger overlap with mercury intrusion characterization scale.

### Experimental results

#### SANS data

**Figure 5:**
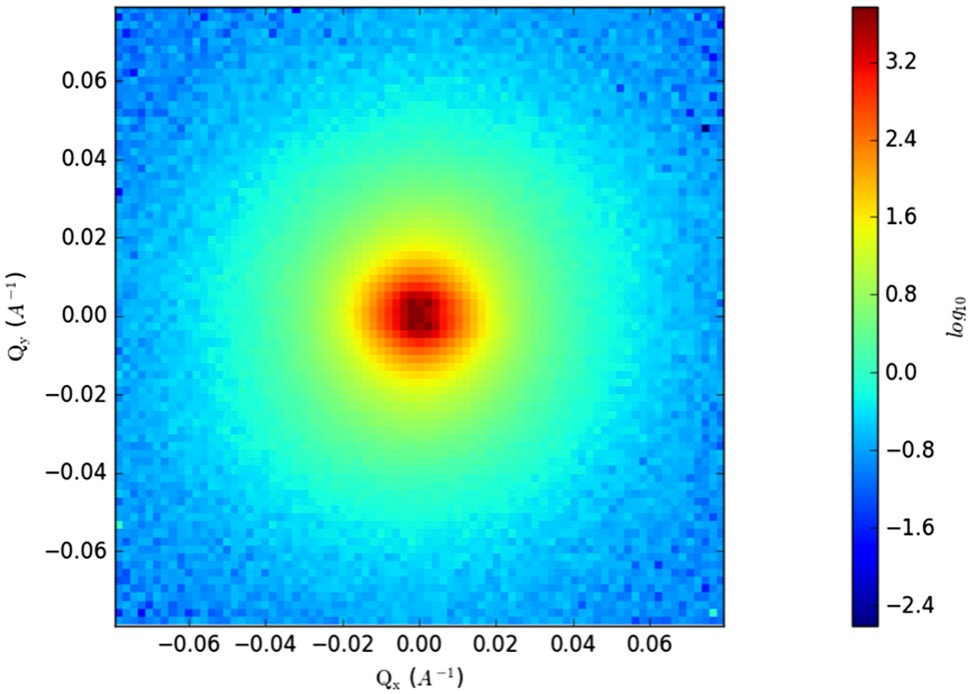
Two dimensional spectrum.

The two-dimensional raw data has been deducted by the background scattering of the empty sample pool, and the absolute intensity is one-dimensional by the IGOR Pro software. The data is analyzed by Irena macro plug-in of IGOR Pro software. The samples are all taken parallel to the direction of bedding, which eliminates the problem of orientation. The two-dimensional spectra of typical samples are shown in Fig. 5. One dimensional results are obtained by integrating the two-dimensional spectra.

**Figure 6:**
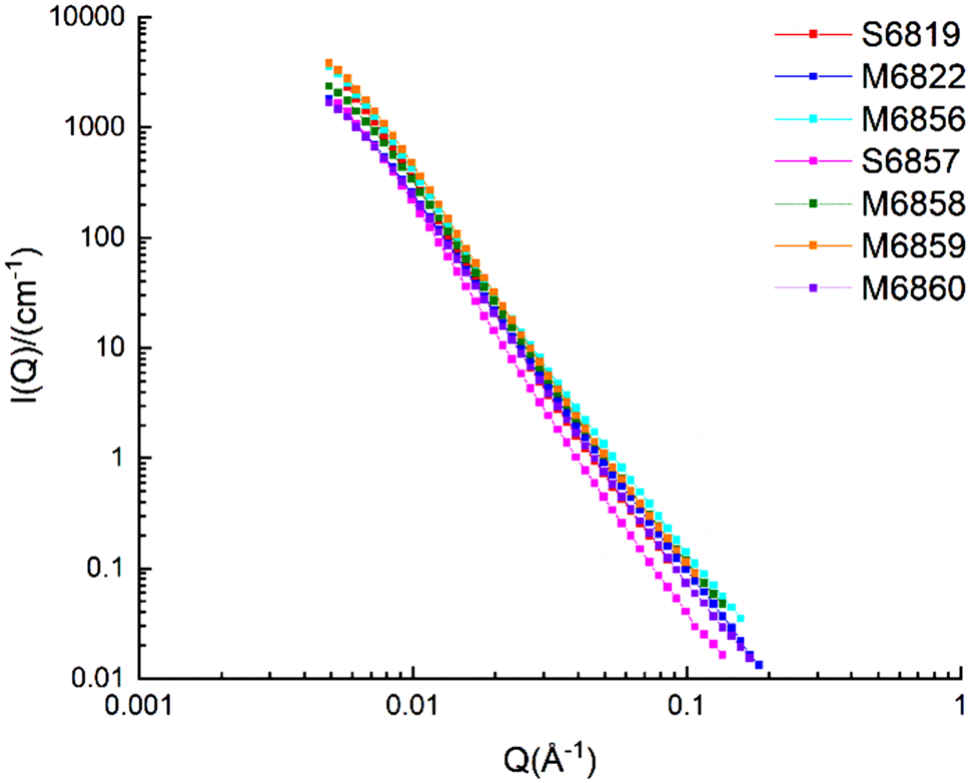
Corrected SANS curve of broad sense shale oil reservoir samples.

The original data of the scattering intensity *I*(*Q*) and the scattering vector *I*(*Q*) can be obtained using the IGOR Pro software. The presence of hydrogen atoms in the sample will cause an incoherent scattering background. The correlation coefficient needs to be adjusted to obtain the value of the horizontal incoherent background of the sample. The SANS curve, after removing the horizontal background, should follow the power law pattern, so the incoherent background value can be subtracted from the scattering intensity *I*(*Q*) in the original data to obtain the corrected scattering intensity *I*(*Q*), and then the correlation between the corrected scattering intensity *I*(*Q*) and scattering vector *Q*. The polydisperse size-distribution model (PDSM) was used to analyze to data. The assumption of the PDSM was that pore network conforms to polydisperse spherical pore network in shale and ignores the contribution from the structure factor term^34,35^. The PSD of samples can be calculated from the polydisperse spheres (PDSM) model in the PRINSAS software. The SANS curve of the sample after PDSM model fitting and removing the horizontal background is shown in Fig. 6.

#### MICP data

**Figure 7:**
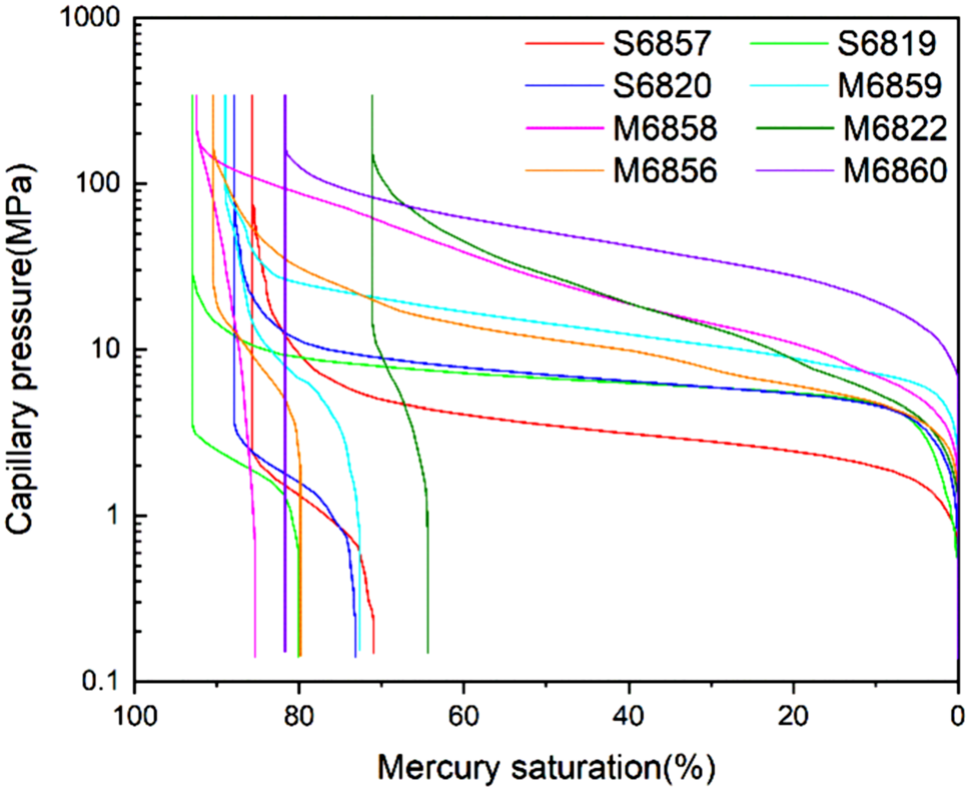
Mercury capillary pressure curves.

The mercury injection and withdrawal curves of the 8 samples were similar in shape. At the initial stage of mercury injection, the injection pressure rises sharply for a short period of time, and then the mercury injection curve gradually reaches a plateau (Fig. 7), indicating that there are extensive macro pores and throats in the samples. The high drainage pressure and low sorting coefficient reflect that the reservoir is characterized by low permeability and yet good sorting.

Both the displacement pressure and the median pressure are negatively correlated with the permeability. The average displacement pressure is 3.15 MPa and the average median pressure is 12.41 MPa. The maximum mercury saturation and mercury withdrawal efficiency of each sample are not much different. The average maximum mercury saturation is 87.50%, and the average mercury withdrawal efficiency is 17.19%, as shown in Table 2. The broad sense shale oil reservoir has high mercury intrusion saturation and low mercury withdrawal efficiency, indicating that the sample has relatively large pores and throats, and good pore-throat connectivity; however the pore-throat configuration is nearly dumbbell-shaped with relatively large pore/throat radius ratios.

**Table 2:** Results of high-pressure MICP tests.

Sample no.	Displacement pressure (MPa)	Median pressure (MPa)	Maximum mercury saturation (%)	Mercury withdrawal efficiency (%)	Sorting coefficient	Skewness
S6857	1.24	3.52	85.7	17.19	0.069	0.18
S6819	2.91	6.57	93.01	13.82	0.039	0.04
S6820	2.88	6.87	87.89	16.67	0.045	0.09
M6859	2.91	6.57	93.01	13.82	0.039	0.04
M6858	2.88	6.87	87.89	16.67	0.045	0.09
M6822	5.43	14.41	88.98	18.34	0.057	0.03
M6856	3.94	26.14	92.45	7.62	0.106	0.09
M6860	2.98	28.32	71.1	9.42	0.105	-0.01
Average	3.15	12.41	87.5	14.19	0.063	0.07

### Analysis and discussion

#### Shapes of full-scale pore size distribution curves

**Figure 8:**
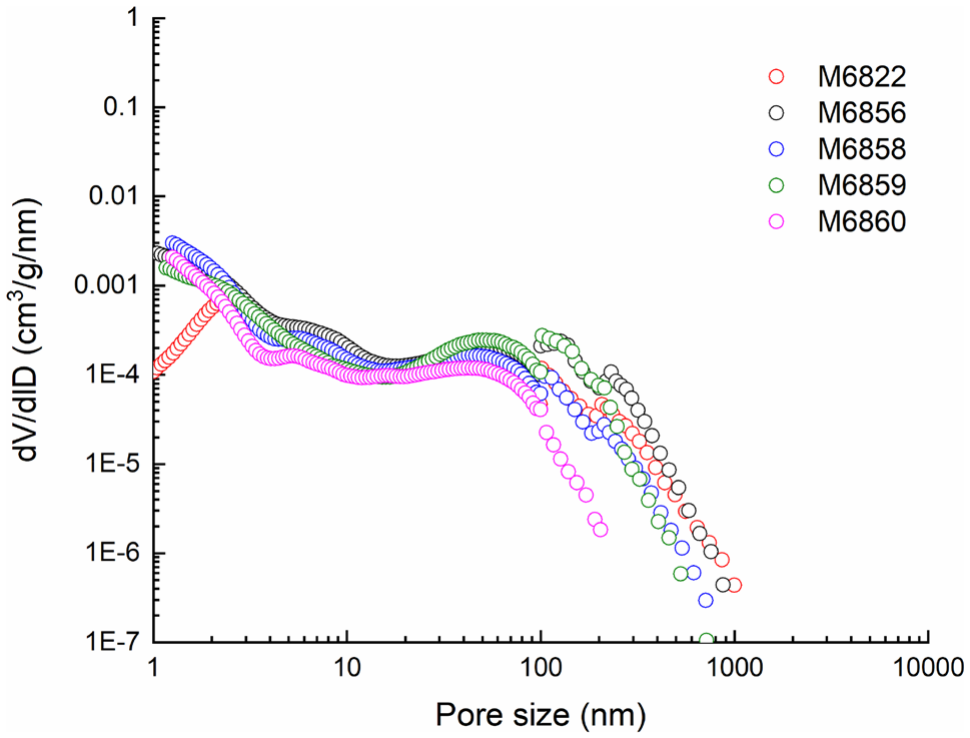
Declining-type pore size distribution curve.

**Figure 9:**
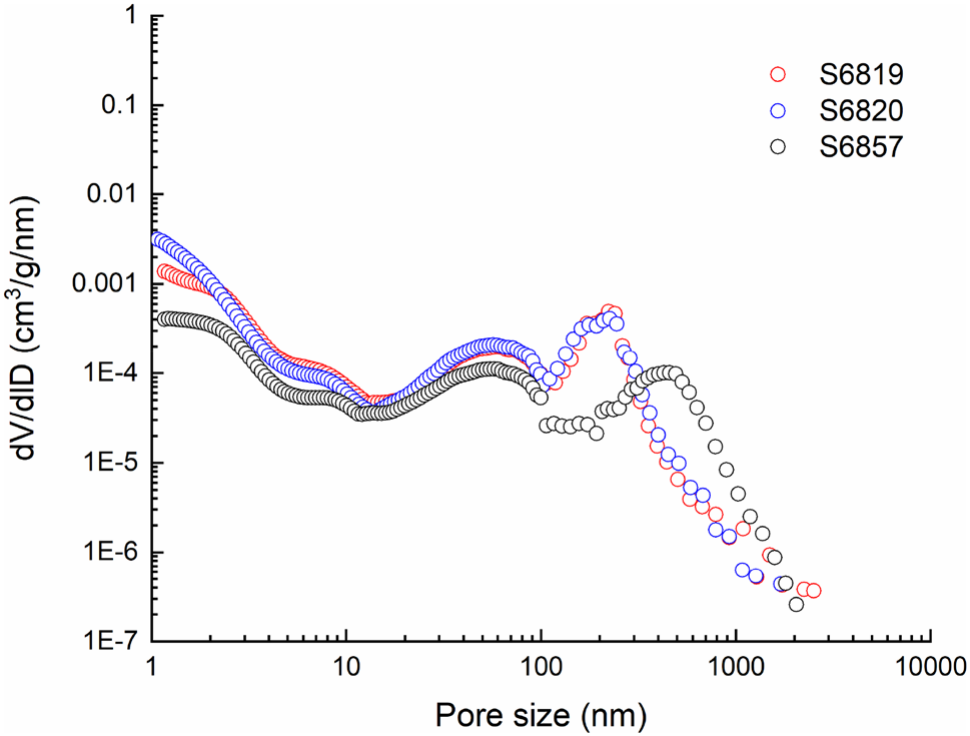
Submicron pore-dominated pore size distribution curve.

The pore size distribution of the Jimsar broad sense shale oil reservoir core samples can be mainly divided into two types: the declining type and submicron pore-dominated type, as shown in Figs. 8 and 9. The submicron pore-dominated type is mostly seen in silt-fine sandstones and dolarenite, with most pores smaller than 500 nm. The declining type is mainly attributed to the silty mudstone and dolomitic mudstone, with most pores less than 80 nm in diameter. The porosities of Samples S6819, S6820 and S6857 are 14.1%, 15.4% and 11.4%, respectively, with an average of 13.6%; those of M6822, M6856, M6858, M6859 and M6860 are 8.6%, 10.4%, 6.0%, 9.3% and 4.5%, respectively, with an average of 7.8%. The results show that the average porosity of the sample with the submicron pore-dominated pore size distribution is about 1.7 times that of the sample with the declining pore size distribution. It has larger specific pore volumes of macropores and a larger average pore size, so the silt-fine sandstone and dolarenite provide the main space for shale oil storage and flow.

#### Quantitative analysis of pores of different scales

According to the international convention, pores with diameters of 0.1–100 nm are defined as nano-scale pores; pores with diameters of 100–1000 nm, submicron pores; pores greater than 1000 nm, micron-scale pores. Samples S6819 and S6820 are of the submicron pore-dominated type. Their average specific pore volume of nano-scale pores is 10.60 mm^3^/g; that of the submicron pores is 59.91 mm^3^/g. The submicron pore dominated type feature large pore space at the submicron scale, and also has a small quantity of micron-scale pores. Samples M6822, M6856 and M6858 are of the declining type, with almost no micron-scale pores. Their average specific pore volume of nano pores is 21.60 mm^3^/g; that of submicron pores is 16.650 mm^3^/g, indicating that the pore space declines as the pore size grows (Table 3).

**Table 3:** Specific pore volume of pores of different scales (unit: mm^3^/g).

Sample no.	Nano pores	Submicron pores	Micron pores
S6819	10.66	58.72	0.91
S6820	10.53	61.09	0.30
M6822	28.53	12.43	0.06
M6856	25.19	29.71	0.00
M6858	11.08	7.81	0.00

**Figure 10:**
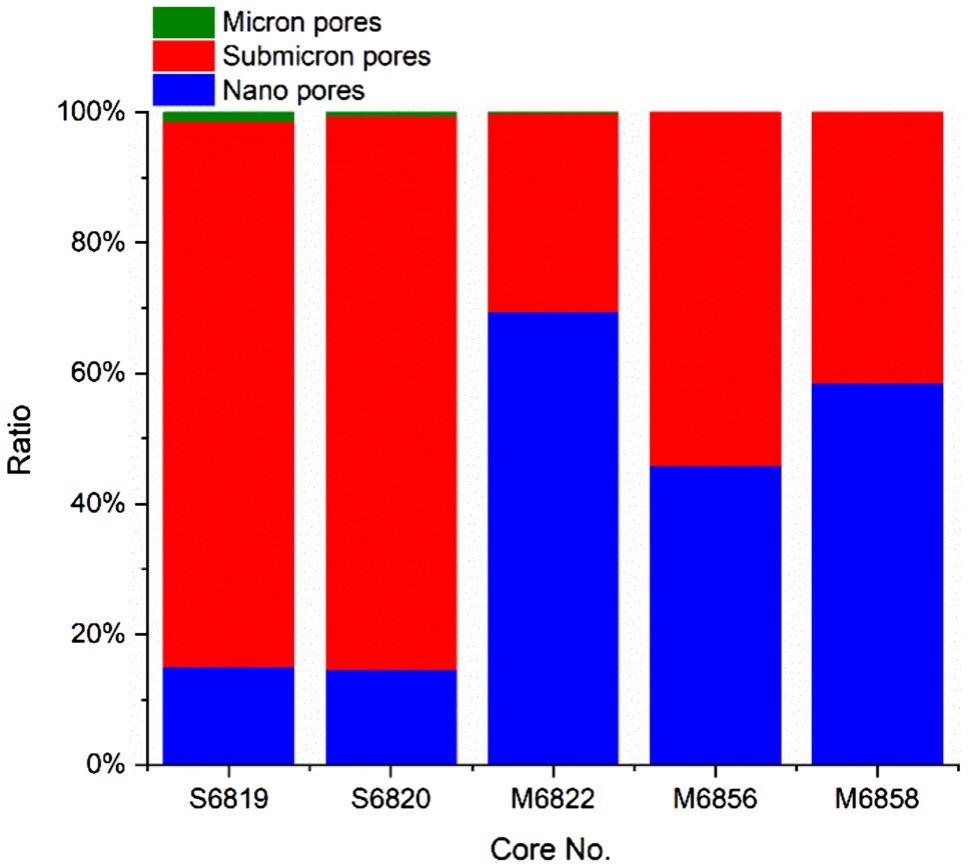
Proportions of pores at different scales.

In view of the average proportions of pores at different levels, about 84% of the total pore space is attributed to the submicron pores in the submicron pore-dominated sample, with about 1% to micron pores and 15% to nano pores. For the declining-type sample, about 60% of the total pores space is contributed by nano pores, with the rest 40% attributed to submicron pores (Fig. 10).

#### Testing method comparison

**Figure 11:**
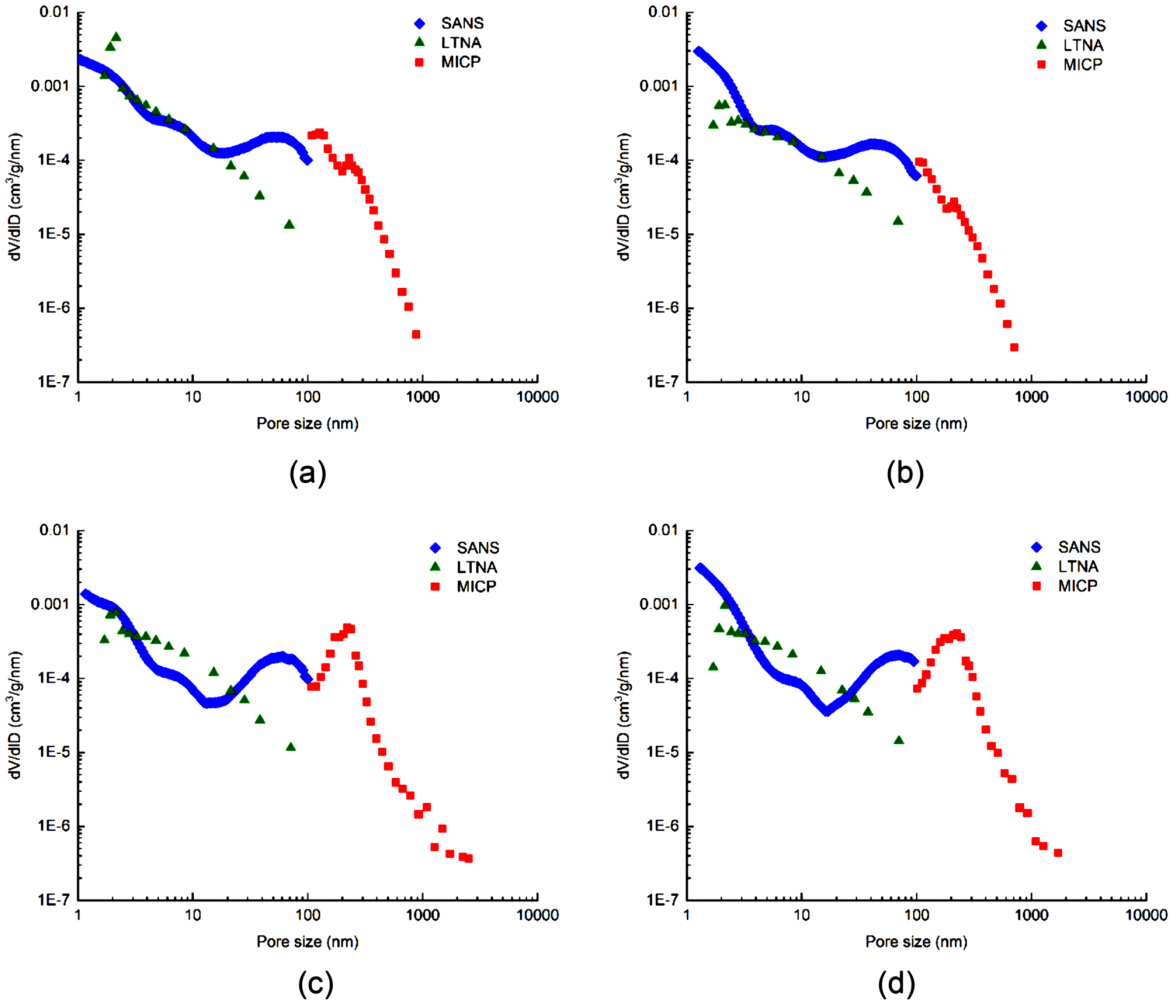
The pore size distribution curves of the three testing methods.

It can be seen from the nano-scale pore size distribution curve measured by SANS that the value of the dV/dlD pore volume at the merging interface (100 nm) has the same order of magnitude with that of submicron pores measured by high-pressure MICP, and the two values are similar. However, the dV/dlD pore volume of nano pores at the merging interface, measured by the low-temperature N_2_ adsorption (LTNA), is about an order of magnitude lower than that measured by high-pressure MICP, as shown in Fig. 11a and b. For the LTNA-MICP full-scale pore size distribution test method, the particle sizes of the prepared samples are quite different. The samples used for LTNA need to be made into 30–100 mesh particles (powder), while the samples used for MICP are usually cylinders with a diameter of 2.5 cm and height of 1 cm. 30–100 mesh particle samples may have destructed pores between 50–100 nm, resulting in the corresponding dV/dlD pore volume of such pores is significantly lower than that measured by SANS, as shown in Fig. 11c and d. The sample used for SANS is fabricated into a 10 mm × 10 mm × 0.5 mm sheet specimen, which is more similar in size to the sample for high-pressure MICP. Moreover, two adjacent parts of one core sample can be tested via SANS and MICP, respectively. Therefore, pore size distribution curve merging between SANS and MICP is better than that of the LTNA-MICP full-scale pore size distribution test, and thus is more applicable to shale oil reservoir samples.

SANS is an effective method for characterization of mesoporous materials, and has been widely used in the shale gas industry over recent years. This study confirms that SANS is an effective and powerful tool for non-destructive characterization of internal structures. In addition, SANS has obvious advantages in characterizing micropores, and MICP is more suitable for investigating macropores. Therefore, the integration of SANS and MICP can not only capture the characteristics of the full-scale pore size distribution, but also improves the accuracy of PSD evaluation. For broad sense shale oil samples, using SANS to measure nano pore size distribution and MICP to measure submicron pore size distribution is a more reasonable method.

## Conclusions

Integration of small-angle neutron scattering (SANS) and mercury intrusion capillary pressure testing (MICP) provides an effective tool for full-scale pore size characterization. The samples measured by SANS and MICP are both in centimeter scale, and there is no pore damage or pore generation during the sample preparation process, which eliminates possible errors in the particle preparation process of the sample used for the gas adsorption method.

The pore size distribution of broad sense shale oil reservoir samples of the Permian Lucaogou Formation in the Jimsar Sag is mainly divided into two types: the declining type and the submicron pore-dominated type. Approximately 84% of the total pore space in the submicron pore-dominated sample is contributed by submicron pores. About 60% of the total pore space in the declining-type sample is contributed by nano pores.

The declining type is mainly silty mudstone and dolomitic mudstone, and most of its pores are smaller than 80 nm. Silt-fine sandstone and dolarenite are mostly of the submicron pore-dominated type, with most pores smaller than 500 nm, and high specific pore volume and average pore diameter of macropores. They are favorable lithogeneous phase for development of high-quality reservoirs.
